# Laryngotracheal Stenosis Secondary to Mantle Cell Lymphoma

**DOI:** 10.7759/cureus.61900

**Published:** 2024-06-07

**Authors:** Jose de Jesus Ley-Tomas, Guillermo Missael Perez-Delgadillo, Cecilia Espinosa-Arce, Luis Stefano Ramirez-Gil

**Affiliations:** 1 Otolaryngology - Head and Neck Surgery, Instituto Nacional de Enfermedades Respiratorias, Mexico City, MEX; 2 Otolaryngology - Head and Neck Surgery, Hospital Angeles Metropolitano, Mexico City, MEX; 3 Medicine, Instituto Politécnico Nacional, Mexico City, MEX

**Keywords:** laryngotracheal stenosis, subglottic lymphoma, primary laryngeal lymphomas, laryngeal lymphoma, mantle lymphoma

## Abstract

Acute laryngeal dyspnea is a life-threatening emergency often attributed to laryngeal tumors or inflammatory edema in adults. Primary laryngeal lymphomas are especially infrequent. As an aggressive subtype of B-cell non-Hodgkin lymphoma (NHL), mantle cell lymphoma (MCL) cases are particularly complex.

Herein, we present a case of laryngotracheal stenosis secondary to primary MCL. A comprehensive assessment, including in-office flexible laryngoscopy, revealed distinct findings within the supraglottis and subglottis. In the supraglottis, a submucosal lesion with well-defined, rounded edges was observed, while the subglottis exhibited a friable tumor occupying approximately 90% of the airway. This necessitated immediate intervention, leading to microlaryngoscopy, biopsy, and open tracheostomy. Due to the rarity of primary laryngeal lymphomas, they present a significant diagnostic challenge. Timely diagnosis is crucial to enable tailored therapeutic strategies and improved patient outcomes. This case highlights the importance of considering lymphomatous etiologies in the management of laryngotracheal stenosis and emphasizes the need for a multidisciplinary approach to optimize patient care.

## Introduction

There are multiple causes of laryngotracheal stenosis, one of which is neoplastic. Primary hematologic laryngeal tumors are rare, representing less than 1% of all laryngeal neoplasms [1]. Primary non-Hodgkin lymphomas (NHLs) of the larynx are the second most common hematopoietic tumors after plasmacytoma, with approximately 100 cases described in the English literature [2-4].

We report a case of laryngotracheal stenosis caused by a primary mantle cell lymphoma (MCL) requiring microlaryngoscopy, biopsy, and open tracheostomy. In addition, we conduct a review of key diagnostic features and analogous cases documented in previous literature.

## Case presentation

An 84-year-old Hispanic male patient presented to the Otolaryngology Department with dyspnea on moderate exertion over the prior two months, which six weeks before consultation progressed to dyspnea on mild exertion and stridor. The patient had a history of systemic arterial hypertension, invasive adenocarcinoma of the colon, clear cell renal carcinoma, and prostate adenocarcinoma.

Seven years prior, he was diagnosed with MCL in the eyelid and received treatment with R-mini-CHOP and methotrexate with a full response. A relapse in 2018 was treated with unspecified chemotherapy as well as 22 cycles of radiotherapy and hormone therapy.

Flexible laryngoscopy conducted in-office revealed the following findings: a submucosal lesion within the supraglottis, characterized by well-defined, rounded edges, measuring 1.5 x 2 cm, exhibiting partial obstruction and non-friability. In the subglottis, a friable tumor with indistinct borders was noted, occupying approximately 90% of the airway and precluding assessment of the remainder of the lower airway.

**Figure 1:**
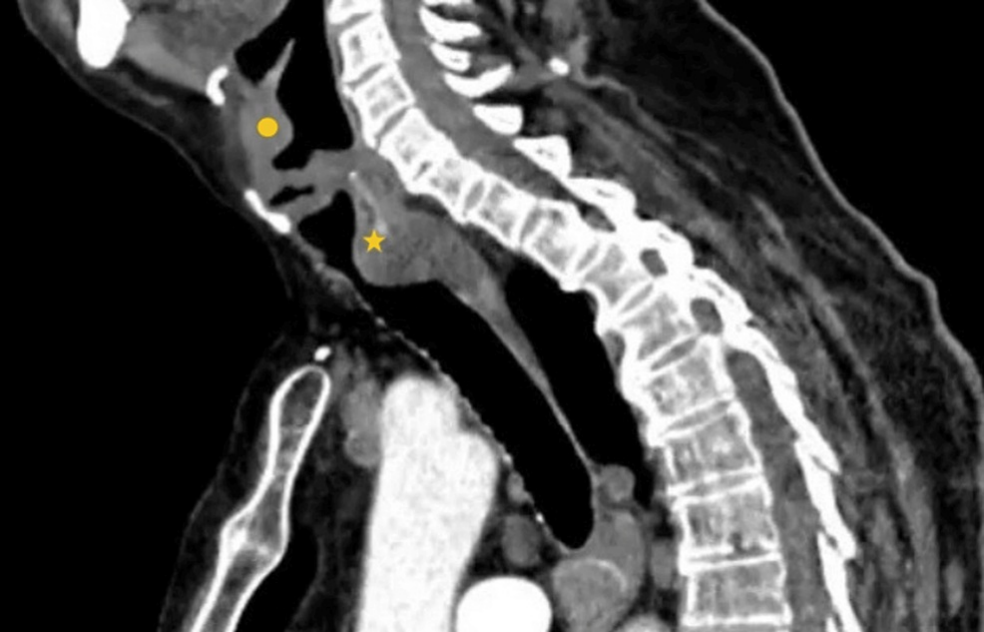
Sagittal CT scan Both masses protrude into the airway, one from the base of the epiglottis on the laryngeal surface, and the other from the posterior part of the subglottic region ⚫: supraglottic mass; ★: subglottic mass

A CT scan (Figure 1) revealed that in the supraglottis, on the laryngeal surface of the epiglottis, a homogeneous soft tissue density with well-defined borders, measuring 6 x 9 mm in diameter, exhibiting expansive growth, and infiltrative characteristics. This density does not enhance with intravenous contrast. Airway obstruction is noted, predominantly involving posterior muscle-like density, extending from the lower third of the cricoid cartilage to approximately the second tracheal ring, leaving a minimal lumen of 6 x 10 mm. There is apparent infiltrative growth with well-defined borders that does not enhance with intravenous contrast.

Due to respiratory instability, it was decided to proceed to the operating room for direct microlaryngoscopy + biopsy + open tracheostomy. These interventions were concluded without further complications and were successful in securing the patient’s airway.

**Figure 2:**
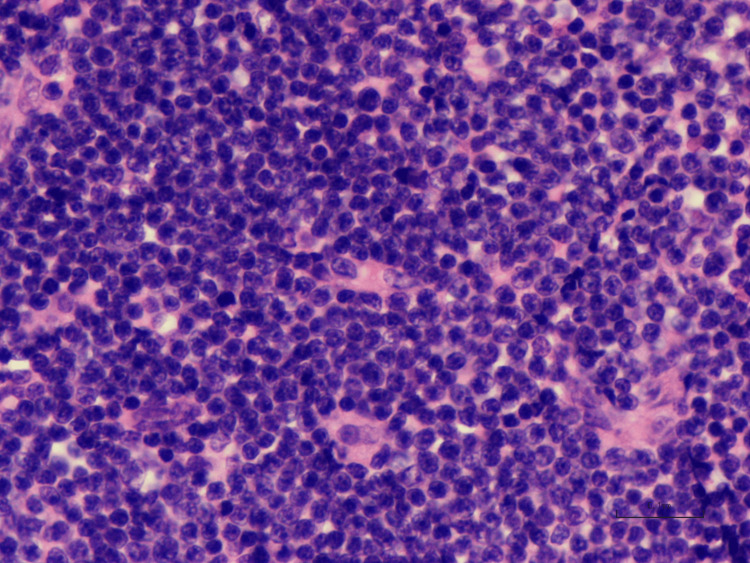
Infiltrate of lymphoid cells on H&E Photomicrograph of a subglottic mass showing uniform small to medium-sized atypical lymphoid cells

During post-surgical evaluation appointments, the biopsy result revealed mantle cell NHL (Figure 2), and immunohistochemistry showed cyclin D1(+), CD20(+), CD5(+), and CD3(+). Since the patient had a secure airway, he was offered medical treatment; however, the patient declined any further testing and refused additional treatment.

## Discussion

We present a case of laryngotracheal stenosis resulting from a subglottic mass, necessitating immediate hospitalization due to compromised airway patency. The pathological examination revealed MCL.

Acute laryngeal dyspnea constitutes a life-threatening emergency and is often attributed to laryngeal tumors or inflammatory edema in adults [5]. Symptoms of laryngeal lymphoma typically include progressive dysphonia, with or without B-symptoms such as fevers, chills, weight loss, and fatigue. Laryngoscopy may reveal a smooth or polypoid, non-ulcerated tumor primarily located in supraglottic structures, such as the epiglottis or aryepiglottic folds. This predilection is associated with a higher concentration of follicular lymphatic tissue compared to the glottis or subglottis [6]. The supraglottic region is most commonly involved (47%), followed by glottic involvement (25%), whereas transglottic and subglottic regions are less frequently affected [7]. 

Primary laryngeal lymphomas pose a diagnostic challenge due to the absence of clinical and gross differential criteria compared to squamous cell carcinoma [8]. Imaging features that are suggestive of laryngeal lymphoma include a large, non-necrotic supraglottic lesion with homogeneous enhancement. Lymphoma may extend into the hypopharynx or remain isolated in the supraglottis, typically lacking calcifications. Similar to squamous cell carcinoma, lymphoma can spread to adjacent structures, such as the subglottis, oropharynx, strap muscles, and laryngeal cartilage. However, cervical lymphadenopathy, which is common in supraglottic squamous cell carcinoma, occurs less frequently in laryngeal lymphoma cases (about 25% of cases) [1,9]. 

MCL is a rare subtype of B-cell NHLs characterized by a t(11;14) chromosomal translocation, resulting in cyclin D1 (CCND1) gene overexpression. Although some cases may be straightforward in their presentation, the morphologic variability can complicate diagnosis. Typically, MCL exhibits an aggressive clinical course, although indolent leukemia variants have been described [10].

MCL accounts for around 5% of all NHLs, with an annual incidence of one case per 200,000 individuals. It occurs more frequently in men than women (three to one), with a median age at diagnosis ranging from 60 to 70 years old [10].

Case reports (Table 1) illustrate varied clinical presentations, ranging from isolated hoarseness to subglottic tumors necessitating tracheotomy. Diagnostic workup typically involves laboratory examinations, including complete blood count (CBC) with differential, lactate dehydrogenase (LDH), and beta-2 microglobulin, alongside imaging (CT or fluorodeoxyglucose-positron emission tomography (FDG-PET)/CT) and bone marrow biopsy. Further diagnostic considerations include the Ki-67 index and mutation status of p53, ataxia-telangiectasia mutated (ATM), and CCND1 [10,11]. CSF studies are recommended to rule out CNS involvement, especially in patients with high Ki-67 index, blastoid variants, or neurologic symptoms [10,11]. Chemoimmunotherapy remains the conventional front-line therapy for MCL [10]. Surgery is not considered an option for these tumors, but conservative biopsy with limited surgical resection may alleviate acute airway obstruction [12].

**Table 1:** Case reports of mantle cell lymphoma of the larynx in recent years M: male; F: female

Article	Age (years)	Gender	Clinical presentation	Tracheotomy	Location
Naciri et al. [13]	70	M	Progressive dyspnea	Yes	Subglottis
Kumbul et al. [14]	76	M	Progressive nasal obstruction	No	Nasopharynx and left aryepiglottic fold
Ababou et al. [15]	43	F	Bradypnea	Yes	Subglottis
Bernstein et al. [12]	71	M	Progressive dysphagia, dysphonia, and dry cough	No	Left aryepiglottic fold and false vocal fold
Groom et al. [16]	60	M	Progressive hoarseness	No	Left false vocal cord

## Conclusions

The management of laryngotracheal stenosis underscores the critical importance of ensuring airway patency. While maintaining respiratory function is paramount, biopsy emerges as a pivotal diagnostic tool, directing us toward the underlying etiology and facilitating tailored therapeutic interventions that may mitigate disease progression and dissemination.
